# Inference of biological networks using Bi-directional Random Forest Granger causality

**DOI:** 10.1186/s40064-016-2156-y

**Published:** 2016-04-26

**Authors:** Mohammad Shaheryar Furqan, Mohammad Yakoob Siyal

**Affiliations:** INFINITUS, Infocomm Centre of Excellence, Nanyang Technological University, Singapore, Singapore; School of Electrical and Electronics Engineering, Nanyang Technological University, Singapore, Singapore

**Keywords:** Biological network, Brain connectivity, Gene network, Random forest, Granger causality

## Abstract

The standard ordinary least squares based Granger causality is one of the widely used methods for detecting causal interactions between time series data. However, recent developments in technology limit the utilization of some existing implementations due to the availability of high dimensional data. In this paper, we are proposing a technique called Bi-directional Random Forest Granger causality. This technique uses the random forest regularization together with the idea of reusing the time series data by reversing the time stamp to extract more causal information. We have demonstrated the effectiveness of our proposed method by applying it to simulated data and then applied it to two real biological datasets, i.e., fMRI and HeLa cell. fMRI data was used to map brain network involved in deductive reasoning while HeLa cell dataset was used to map gene network involved in cancer.

## Background

The concept of causal influence can be dated back in 1956 when Wiener (1956) conceived idea that if including the information of one time series can improve the prediction of other time series, this means that the second series has a causal influence on the other. After more than a decade, the same concept was practically formalized by Granger (1969) in 1969, for studying the causal interaction between financial time series data. Moreover, recently the idea of Granger causality has also been utilized in bio-informatics for studying brain connectivity map (Ding et al. 2006; Hu and Liang 2014; Lang et al. 2012; Liao et al. 2011), gene networks (Michailidis and d’Alche-Buc 2013; Tam et al. 2012), and more.

However, with the advancement in technology, data acquisition techniques can now simultaneously analyze multiple variables and produce high-dimensional data, and since Granger uses ordinary least squares (OLS) method for evaluating Granger causality, it is not a viable option when it comes to handling high dimensional data. The reason for this limitation is the fact that the OLS application requires less number of variables compared to observational time points. Therefore, in order to resolve this limitation, several alternates were discussed in the past that includes the use of other regularization techniques (Shojaie and Michailidis 2010; Tang et al. 2012; Valdés-Sosa et al. 2005), kernel-based methods (Liu et al. 2014; Marinazzo et al. 2008) and neural network based methods (Montalto et al. 2015).

Recently, two viable options were discussed by Furqan and Siyal (2015) and Cheng et al. (2014). Furqan and Siyal (2015) proposed to use Random Forest as a regularization technique for evaluating Granger causality whereas Cheng et al. (2014) proposed an LASSO-based method to reuse the time series data by reversing the time stamp of the time series. This concept of time reversal is also discussed and used by other researchers including Haufe et al. (2012), Hu et al. (2015) and others.

In this paper, we are proposing an improved method based on a combination of Random Forest Granger causality and re-utilization of time series data. We are calling it Bi-directional Random Forest Granger causality. This proposed method has increased precision and efficiency compared to existing LASSO-based method proposed by Cheng et al. (2014). In order to provide the proof of improvements of our method, we applied these methods to simulated data before mapping two different real biological networks i.e., gene and brain network.

## Methods

### Random Forest Granger causality

**Fig. 1:**
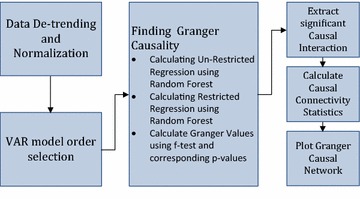
Ray diagram to implement Random Forest Granger causality as proposed by Furqan and Siyal (2015)

Random Forest is a decision tree based learning algorithm that was initially proposed by Breiman (2001) as a classification technique. However, later Liaw and Wiener (2002) suggested that Random Forest can also be used as regularization technique. This proposition of Liaw and Wiener (2002) to use Random Forest as a regularization technique was discussed and applied by Furqan and Siyal (2015) for evaluating coefficients of vector autoregressive model. They have performed Rigorous experimentations to prove its effectiveness. Its implementation follows the ray diagram shown in Fig. 1.

### Naïve Forward Backward LASSO Granger causality

Cheng et al. (2014) proposed Naïve Forward Backward LASSO Granger causality which can handle the shortage of data by reusing the time series data after reversing the time stamp of data. They called this method Naïve Forward Backward LASSO Granger causality. In explaining their proposed method, they use the assumption that the original time series validates all necessary conditions to perform Granger casualty analysis as studied in Bahadori and Liu (2013) and Eichler (2011) and included linearity and stationarity of time series. Once all the conditions are validated, they have proposed to use the pseudo code discussed below that uses LASSO-Based Granger causality analysis algorithm that is available at Bahadori (2014).

### Bi-direction Random Forest Granger causality

Based on the findings of Naïve Forward Backward LASSO Granger Causality and Random Forest Granger causality, we are proposing to use Random Forest Granger causality together with the concept of re-utilization of time series data by reversing the data time stamps in order to maximize advantages in terms of precision, false discovery rate, recall, and F1-score. The pseudo code for evaluating Bi-directional Random Forest Granger causality is as follow:

## Experimental details

We have implemented the basic Random Forest method on MATLAB with the help of R package (Breiman 2001). Later, we merged the implemented code with Granger causality analysis (GCCA) toolbox (Seth 2010) for evaluating Granger causality that uses BSMART toolbox (Cui et al. 2008). Whereas, we have used Akaike Information Criterion (AIC) as discussed by Akaike (1974) for VAR model order selection.

After the implementation of proposed method, we have compared our method with Cheng et al. (2014), LASSO-based method. Cheng’s method, using four measures: precision, false discovery rate, recall, and F1-score. These measures were evaluated against ground truth network shown in Fig. 5 using the following mathematical equations:

$$\begin{aligned} & Precision = \frac{True\,positive\,edges}{True\,positive\,edges + False\,positive\,edges}. \\ & Recall = \frac{True\,positive\,edges}{True\,positive\,edges + False\,negative\,edges} \\ & F1{\text{-}}Score = \frac{2 \times True\,positive\,edges}{{\left( {2 \times True\,positive\,edges} \right) + False\,positive\,edges + False\, negative\,edges}} \\ \end{aligned}$$

### Simulated network

**Fig. 2:**
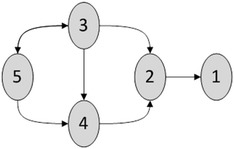
Ground Truth Network of five variable simulated dataset

In order to remain unbiased in our comparative study, we utilized a simulated network dataset that has been previously used by researchers like Furqan and Siyal (2015), Schelter et al. (2006), and more. The simulated data set simulates five variable scenarios. Its ground truth network is shown in Fig. 2, and its network can be modeled using following mathematical equations:

$$\begin{aligned} x_{1} \left( t \right) & = 0.6 x_{1} \left( {t - 1} \right) + 0.65 x_{2} \left( {t - 2} \right) + \varepsilon_{1} \left( t \right) \\ x_{2} \left( t \right) & = 0.5 x_{2} \left( {t - 1} \right) - 0.3 x_{2} \left( {t - 2} \right) - 0.3x_{3} \left( {t - 4} \right) + 0.6 x_{4} \left( {t - 1} \right) + \varepsilon_{2} \left( t \right) \\ x_{3} \left( t \right) & = 0.8 x_{3} \left( {t - 1} \right) - 0.7 x_{3} \left( {t - 2} \right) - 0.1 x_{5} \left( {t - 3} \right) + \varepsilon_{3} \left( t \right) \\ x_{4} \left( t \right) & = 0.5 x_{4} \left( {t - 1} \right) + 0.9 x_{3} \left( {t - 2} \right) + 0.4 x_{5} \left( {t - 2} \right) + \varepsilon_{4} \left( t \right) \\ x_{5} \left( t \right) & = 0.7 x_{5} \left( {t - 1} \right) - 0.5 x_{5} \left( {t - 2} \right) - 0.2 x_{3} \left( {t - 1} \right) + \varepsilon_{5} \left( t \right) \\ \end{aligned}$$

where ɛ_1_(t), ɛ_2_(t), ɛ_3_(t), ɛ_4_(t), and ɛ_5_(t) are independent and identically distributed white noise with E(ɛ_1_(t)) = E(ɛ_2_(t)) = E(ɛ_3_(t)) = E(ɛ_4_(t)) = E(ɛ_5_(t)) = 0, E(ɛ_1_(t)ɛ_1_(t)′) = E(ɛ_2_(t)ɛ_2_(t)′) = E(ɛ_3_(t)ɛ_3_(t)′) = E(ɛ_4_(t)ɛ_4_(t)′) = E(ɛ_5_(t)ɛ_5_(t)′) = ɛ.

### Real fMRI dataset

In this paper, we have utilized StarPlus data set which was collected to study the working of the brain related to human deductive reasoning. This StarPlus dataset was collected by Keller et al. (2001) and can be freely accessed from Mitchell and Wang (2001).

In this dataset, they had studied 13 normal subjects using 40 trials on each subject. Each trial consists of two major egments. In one segment of the trial, the subject was presented with a visual stimulus in the form of Image for 4 s followed by a 4-s blank screen. Then, in next segment, another visual stimulus was presented for another 4-s in the form of a sentence wich may or may not be related to the image. This visual stimulus was followed by 4-s blank screen. After both stimuli, the subject was asked to decide the presence of a relation between image and sentence. Moreover, each subject was allowed to rest for 15-s before the start of next trial.

In order to introduce randomness in the experiment, 40 trials were divided into two parts of 20 trials each. In 20 trials, subjects were shown image first and then the sentence whereas for remaining 20 trials, they reversed the order of image and sentence. Further information related to experiment settings, sentences, and picture, are explicitly not discussed here and can be referred to Keller et al. (2001).

While performing these trials, T2-weighted fMRI images were collected using 3T Signa scanner at an interval of 500 ms, and with TE = 18 ms and flip angle of 50°. These settings yield images that have approximately 5000 voxels per subjects in 8 oblique axial slices in two different non-contiguous four-slice volumes. The first volume set captures prefrontal areas and superior parietal regions, while, another volume set covers posterior temporal, inferior frontal and occipital areas.

After acquiring T2-weighted fMRI images for each subject, images were pre-processed using FIASCO program (Eddy et al. 1999). This pre-processing helps in reducing the artifacts that arise during image acquisition process due to signal drift, head motion, and others.

After pre-processing of images, 25 anatomical regions of interest were selected that includes left dorsolateral prefrontal cortex (LDLPFC) and right dorsolateral prefrontal cortex (RDLPFC), calcarine sulcus (CALC), left frontal eye fields (LFEF), right frontal eye fields (RFEF), left inferior parietal lobule (LIPL), right inferior parietal lobule (RIPL), left intraparietal sulcus (LIPS), right intraparietal sulcus (RIPS), left inferior frontal gyrus (LIFG), left opercularis (LOPER), right opercularis (ROPER), supplementary motor areas (SMA), left and right inferior temporal lobule (LIT, RIT), left and right posterior precentral sulcus (LPPREC, RPPREC), left and right supramarginal gyrus (LSGA, RSGA), left temporal lobe (LT), right temporal lobe (RT), left and right triangularis (LTRIA, RTRIA), left superior parietal lobule (LSPL) and right superior parietal lobule (RSPL). However, we have restricted our study to 7 regions of interests (ROIs) that were used and advised to be more relevant by other researchers (Furqan and Siyal 2015; Wang and Mitchell 2002) and include LIPL, LDLPFC, CALC, LTRIA, LT, LOPER, and LIPS.

### Real Hela dataset

The HeLa human cancer cell line dataset used in our study was compiled by Whitfield et al. (2002) by performing series of experiments using DNA microarray technique. These experimental results are freely available (Whitfield et al. 2000).

In our study, we have used their experiment 3 dataset to prove more effectiveness of our method as other researchers have commonly used this dataset as well (Hlavácková-Schindler and Bouzari 2013; Lozano et al. 2009). The Experiment 3 dataset has recognized more than 1100 genes that are intermittently expressed during the cancer cell cycle. Based on the recommendations of other researchers (Hlavácková-Schindler and Bouzari 2013; Ogutu et al. 2012), we have used 19 preselected genes that are: PCNA, NPAT, E2F1, CCNE1, CDC25A, CDKN1A, BRCA1, CCNF, CCNA2, CDC20, STK15, BUB1B, CKS2, CDC25C, PLK1, CCNB1, CDC25B, TYMS, and DHFR.

As the observational points are not homogeneously sampled, the data was first interpolated by using cubic smoothing splines (Green and Silverman 1994) as recommended by Hlavácková-Schindler and Bouzari (2013) and Ogutu et al. (2012) before using in our study.

## Results and discussion

### Simulated dataset

**Fig. 3:**
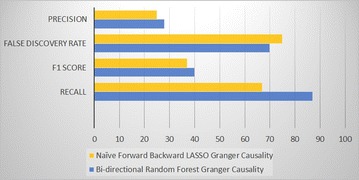
Results of five variable simulated datasets

Based on the results of simulated studies shown in Fig. 3, we found that LASSO-based Forward Backward Granger causality on average yields approximately 25 % precision, 75 % false discovery rate, 67 % recall and 37 % F1 score. Whereas using the same set of data, our proposed method yields 28 % precision, 70 % false discovery rate, 87 % recall, and 40 % F1 score.

These findings suggest that our proposed method has outperformed the existing method in all measures, with a significant improvement in recall. Our proposed method shows 20 % improvement in recall compared to existing LASSO-based method.

During this study, we have observed that the proposed method is less prone to outliers compared to the LASSO-based method. This ability of insensitivity of outlier is achieved due to inherent advantage of regularized tree methods. We have also observed that the proposed method is highly dependent on selecting the right number of features and number of trees. In this study, we have used the setting of 10 features and 500 trees. However, further studies are required to devise some ideal relationship between both number features and number of trees.

### HeLa cell dataset

**Fig. 4:**
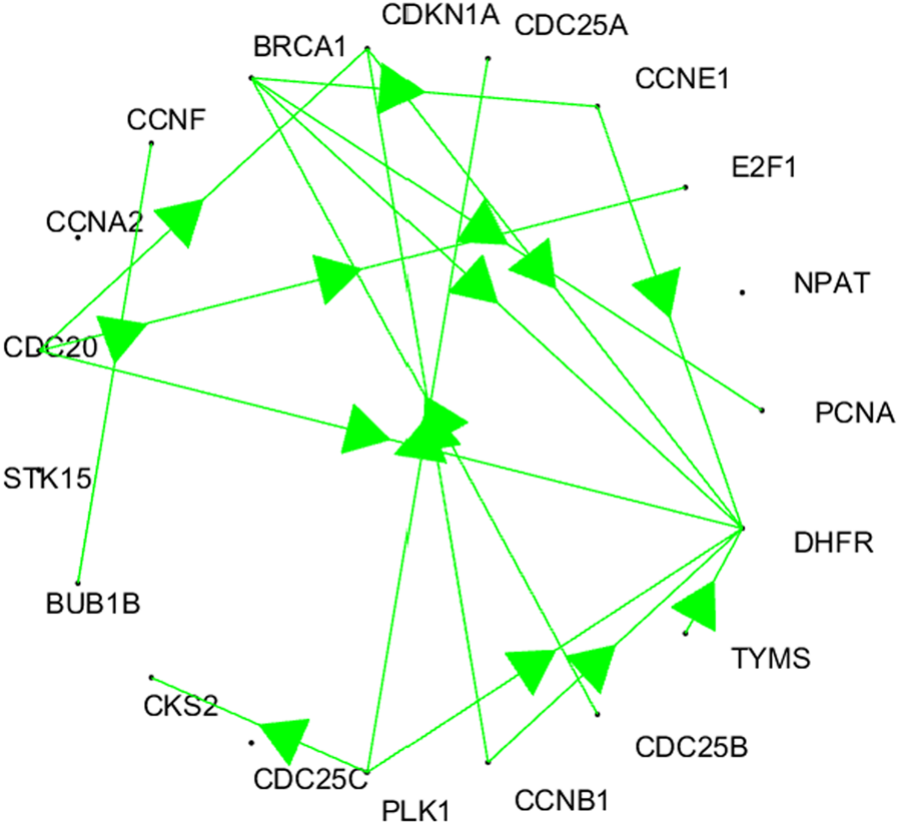
Gene Network found using Bi-directional Random Forest Granger causality

Following the findings of simulated data set studies, we have applied the proposed method to real HeLa cell dataset. The resultant gene network that is involved in cancers is shown in Fig. 4 where the green arrow shows a uni-directional link between two nodes.

As there is no way to verify the resultant network, we have used Biological General Repository for Interaction Datasets BIOGRID database (Chatr-aryamontri et al. 2014) to look for genes interactions that were already reported. The BIOGRID is a public database that archives and disseminates genetic and protein interaction data from model organisms and humans. Given the above network map, we were able of find 6 out 16 interactions that yield around 37 % precision and 63 % false discovery rate. These statistics are in line with the results of the simulated dataset where BRFGC produces 28 % precision and 63 % false discovery rate.

### StarPlus fMRI dataset

**Fig. 5:**
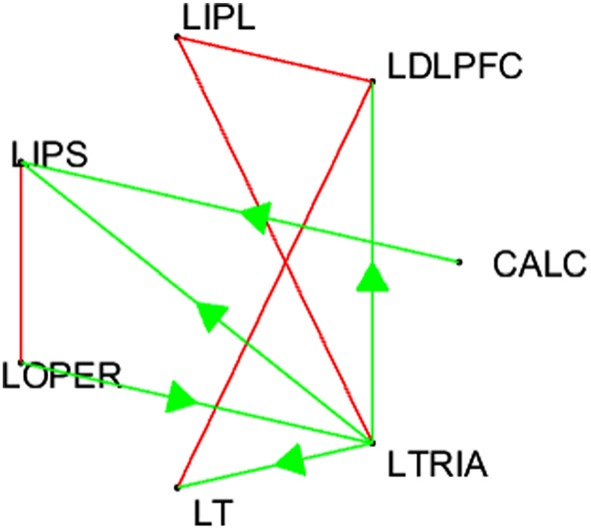
Effective Brain Connectivity map for seven ROIs that are involved in deductive reasoning

For discussing results of real StarPlus dataset shown in Fig. 5, let’s first overview the functions of the pre-selected regions studied in this paper. The first region under consideration is calcarine sulcus (CALC). CALC consist of calcarine cortex that maps the point-to-point representation from the retina to the cortex as discussed by Meadows (2011). The next region under consideration is left intraparietal sulcus (LIPS). This region of the brain is associated with the processing of light contrast elements seen by eyes without analyzing the relationship between those elements (Smith et al. 2014).

Other regions of interest are left opercularis (LOPER) and left triangularis (LTRIA) which are also called Brodmann Area 44 and Brodmann Area 45 (Nishitani et al. 2005), and together they constitute Broca’s region. The Broca’s region is associated with the processing of words, pseudo-words, and non-words during different parts of reading and their interaction as discussed in Heim et al. (2005).

Left dorsolateral prefrontal cortex (LDLPFC) is associated with manipulation of auditory and spatial information in working memory (Barbey et al. 2013) whereas left inferior parietal lobule (LIPL) is necessary for comparison (Chochon et al. 1999), memory related to motor processes (e.g., movement of hand), mechanical and technical reasoning associated with the use of objects (van Elk 2014) and more. Whereas, the remaining region under consideration is left Temporal Lobe (LT) which is mainly associated with the primary organization of sensory inputs (Read 1981).

Based on the functional knowledge of regions of interests, our resulted network in Fig. 3 shows that the connection between CALC with LIPS seems to transfer visual information (picture or sentence displayed on screen), the bi-direction link between LOPER and LIPS signifies the feed-backed link for recognizing the objects and words. The connection between Brodmann area 44 and 45 shows the movement of information from area 44 to area 45 for further processing of information.

The other links such as the links from Brodmann area 45 represents the transfer of information to and from LDLPFC, LIPL and LT for further processing to evaluate the meaning, relation and deduction of the task performed. The remaining bidirectional link between LIPL ↔ LDLPFC and LT ↔ LDLPFC exchange information related to the movement to finger for registering the answer to the task.

## Conclusion

In this paper, we have proposed an improved method called Bi-directional Random Forest Granger causality. It takes the advantage of Random Forest regularization to handle dimensionality issues and at the same time using reversing time stamping property it limits the data shortage problem. Using simulated dataset we have shown the effectiveness of our proposed method and later, we have applied the proposed approach to real StarPlus fMRI data set to study the network involved in human deductive reasoning and to real HeLa cell dataset to map gene network that is involved in cancer. In future, this method can be used in other areas such as econometrics, and social networking.

## References

[CR1] Akaike H (1974). A new look at the statistical model identification. IEEE Trans Autom Control.

[CR2] Bahadori MT (2014) Lasso-Granger. http://www-scf.usc.edu/~mohammab/codes/codes.html

[CR3] Bahadori MT, Liu Y (2013) An examination of practical granger causality inference. Paper presented at the 2013 SIAM international conference on data mining, Austin, Texas, USA

[CR4] Barbey AK, Koenigs M, Grafman J (2013). Dorsolateral prefrontal contributions to human working memory. Cortex.

[CR5] Breiman L (2001). Random forests. Mach Learn.

[CR6] Chatr-Aryamontri A, Breitkreutz BJ, Oughtred R, Boucher L, Heinicke S, Chen D, Stark C, Breitkreutz A, Kolas N, O'Donnell L, Reguly T, Nixon J, Ramage L, Winter A, Sellam A, Chang C, Hirschman J, Theesfeld C, Rust J, Livstone MS, Dolinski K, Tyers M (2015). The BioGRID interaction database: 2015 update. Nucleic Acids Res.

[CR7] Cheng D, Bahadori MT, Liu Y (2014) FBLG: a simple and effective approach for temporal dependence discovery from time series data. Paper presented at the Proceedings of the 20th ACM SIGKDD international conference on knowledge discovery and data mining, New York, USA

[CR8] Chochon F, Cohen L, Van De Moortele P, Dehaene S (1999). Differential contributions of the left and right inferior parietal lobules to number processing. J Cogn Neurosci.

[CR9] Cui J, Xu L, Bressler SL, Ding M, Liang H (2008). BSMART: a Matlab/C toolbox for analysis of multichannel neural time series. Neural Netw.

[CR10] Ding M, Chen Y, Bressler SL (2006). Granger causality: basic theory and application to neuroscience handbook of time series analysis.

[CR11] Eddy WF, Fitzgerald M, Genovese C, Lazar N, Mockus A, Welling J (1999). The challenge of functional magnetic resonance imaging. J Comput Graph Stat.

[CR12] Eichler M (2011). Graphical modelling of multivariate time series. Probab Theory Relat Fields.

[CR13] Furqan MS, Siyal MY (2015). Random Forest Granger causality for detection of effective brain connectivity using high dimensional data. J Integr Neurosci.

[CR14] Granger CWJ (1969). Investigating causal relations by econometric models and cross-spectral methods. Econometrica.

[CR15] Green PJ, Silverman BW (1994). Nonparametric regression and generalized linear models: a roughness penalty approach.

[CR16] Haufe S, Nikulin VV, Nolte G (2012) Alleviating the influence of weak data asymmetries on granger-causal analyses. In: Theis F, Cichocki A, Yeredor A, Zibulevsky M (eds) Latent variable analysis and signal separation: 10th international conference, LVA/ICA 2012, Tel Aviv, Israel, March 12–15, 2012. Proceedings. Springer, Berlin, pp 25–33

[CR17] Heim S, Alter K, Ischebeck AK, Amunts K, Eickhoff SB, Mohlberg H, Zilles K, von Cramon DY, Friederici AD (2005). The role of the left Brodmann’s areas 44 and 45 in reading words and pseudowords. Cogn Brain Res.

[CR18] Hlavácková-Schindler K, Bouzari H (2013) Granger Lasso Causal Models in higher dimensions-application to gene expression regulatory networks. ECML/PKDD 2013 workshop scalable decision making: uncertainty, imperfection, deliberation (SCALE)

[CR19] Hu M, Liang H (2014). A copula approach to assessing Granger causality. NeuroImage.

[CR20] Hu M, Li W, Liang H (2015). A copula-based Granger causality measure for the analysis of neural spike train data. IEEE/ACM Trans Comput Biol Bioinf.

[CR21] Keller TA, Just MA, Stenger VA (2001) Reading span and the time-course of cortical activation in sentence-picture verification. Paper presented at the annual convention of the Psychonomic Society, Orlando, FL

[CR22] Lang EW, Tomé AM, Keck IR, Górriz-Sáez JM, Puntonet CG (2012). Brain connectivity analysis: a short survey. Comput Intell Neurosci.

[CR23] Liao W, Ding J, Marinazzo D, Xu Q, Wang Z, Yuan C, Zhang Z, Lu G, Chen H (2011). Small-world directed networks in the human brain: multivariate Granger causality analysis of resting-state fMRI. NeuroImage.

[CR24] Liaw A, Wiener M (2002). Classification and regression by randomForest. R News.

[CR25] Liu J, Xu Y, Cheng J, Zhang Z, Wong D, Yin F, Wong T, Zhang Y-T (2014). Multiple modality fusion for glaucoma diagnosis. The international conference on health informatics.

[CR26] Lozano AC, Abe N, Liu Y, Rosset S (2009). Grouped graphical Granger modeling for gene expression regulatory networks discovery. Bioinformatics.

[CR27] Marinazzo D, Pellicoro M, Stramaglia S (2008). Kernel-Granger causality and the analysis of dynamical networks. Phys Rev E.

[CR28] Meadows M-E, Kreutzer J, DeLuca J, Caplan B (2011). Calcarine cortex. Encyclopedia of clinical neuropsychology.

[CR29] Michailidis G, d’Alche-Buc F (2013). Autoregressive models for gene regulatory network inference: sparsity, stability and causality issues. Math Biosci.

[CR30] Mitchell T, Wang W (2001) StarPlus fMRI data. http://www.cs.cmu.edu/afs/cs.cmu.edu/project/theo-81/www/

[CR31] Montalto A, Stramaglia S, Faes L, Tessitore G, Prevete R, Marinazzo D (2015). Neural networks with non-uniform embedding and explicit validation phase to assess Granger causality. Neural Netw.

[CR32] Nishitani N, Schürmann M, Amunts K, Hari R (2005). Broca’s region: from action to language. Physiology.

[CR33] Ogutu JO, Schulz-Streeck T, Piepho HP (2012). Genomic selection using regularized linear regression models: ridge regression, lasso, elastic net and their extensions. BMC Proc.

[CR34] Read DE (1981). Solving deductive-reasoning problems after unilateral temporal lobectomy. Brain Lang.

[CR35] Schelter B, Winterhalder M, Eichler M, Peifer M, Hellwig B, Guschlbauer B, Lücking CH, Dahlhaus R, Timmer J (2006). Testing for directed influences among neural signals using partial directed coherence. J Neurosci Methods.

[CR36] Seth AK (2010). A MATLAB toolbox for Granger causal connectivity analysis. J Neurosci Methods.

[CR37] Shojaie A, Michailidis G (2010). Discovering graphical Granger causality using the truncating lasso penalty. Bioinformatics.

[CR38] Smith KW, Vartanian O, Goel V (2014). Dissociable neural systems underwrite logical reasoning in the context of induced emotions with positive and negative valence. Front Hum Neurosci.

[CR39] Tam GHF, Chunqi C, Yeung Sam H (2012, 18–20 Aug. 2012). Application of Granger causality to gene regulatory network discovery. Paper presented at the 2012 IEEE 6th international conference on systems biology (ISB)

[CR40] Tang W, Bressler SL, Sylvester CM, Shulman GL, Corbetta M (2012). Measuring Granger Causality between cortical regions from voxelwise fMRI BOLD signals with LASSO. PLoS Comput Biol.

[CR41] Valdés-Sosa PA, Sánchez-Bornot JM, Lage-Castellanos A, Vega-Hernández M, Bosch-Bayard J, Melie-García L, Canales-Rodríguez E (2005). Estimating brain functional connectivity with sparse multivariate autoregression. Philos Trans R Soc B Biol Sci.

[CR42] van Elk M (2014). The left inferior parietal lobe represents stored hand-postures for object use and action prediction. Front Psychol.

[CR43] Wang X, Mitchell T (2002) Detecting cognitive states using machine learning. Iterim working paper

[CR44] Whitfield ML, Sherlock G, Saldanha A, Murray JI, Ball CA, Alexander KE, Matese JC, Perou CM, Hurt MM, Brown PO, Botstein D (2000) Identification of genes periodically expressed in the human cell cycle and their expression in tumors. http://genome-www.stanford.edu/Human-CellCycle/HeLa/10.1091/mbc.02-02-0030. PMC117619 12058064

[CR45] Whitfield ML, Sherlock G, Saldanha AJ, Murray JI, Ball CA, Alexander KE, Matese JC, Perou CM, Hurt MM, Brown PO, Botstein D (2002). Identification of genes periodically expressed in the human cell cycle and their expression in tumors. Mol Biol Cell.

[CR46] Wiener N, Beckenbach E (1956). The theory of prediction. Modern mathematics for engineers.

